# Serum Thyroid Hormone Antibodies Are Frequent in Patients with Polyglandular Autoimmune Syndrome Type 3, Particularly in Those Who Require Thyroxine Treatment

**DOI:** 10.3389/fendo.2017.00212

**Published:** 2017-08-28

**Authors:** Roberto Vita, Maria Giulia Santaguida, Camilla Virili, Maria Segni, Marina Galletti, Mattia Mandolfino, Flavia Di Bari, Marco Centanni, Salvatore Benvenga

**Affiliations:** ^1^Department of Clinical and Experimental Medicine, University of Messina, Messina, Italy; ^2^Department of Medico-Surgical Sciences and Biotechnologies, Sapienza University of Rome, Latina, Italy; ^3^Department of Pediatrics and Child Neuropsychiatry, Sapienza University of Rome, Rome, Italy; ^4^Endocrinology Unit, AUSL Latina, Latina, Italy; ^5^Master Program on Childhood, Adolescent and Women’s Endocrine Health, University of Messina, Messina, Italy; ^6^Interdepartmental Program of Molecular & Clinical Endocrinology, and Women’s Endocrine Health, University Hospital Policlinico Universitario G. Martino, Messina, Italy

**Keywords:** thyroid hormone antibodies, chronic autoimmune thyroiditis, chronic atrophic gastritis, non-segmental vitiligo, celiac disease

## Abstract

Polyglandular autoimmune syndrome (PAS) type 3 consists of autoimmune thyroid disease (AITD) coexisting with ≥1 non-thyroidal autoimmune disease (NTAID) other than Addison’s disease and hypoparathyroidism. We evaluated the prevalence and repertoire of thyroid hormones antibodies (THAb) in PAS-3 patients. Using a radioimmunoprecipation technique, we measured THAb (T3IgM, T3IgG, T4IgM, and T4IgG) in 107 PAS-3 patients and 88 controls (patients with AITD without any NTAID). Based on the selective coexistence of AITD with one NTAID (chronic autoimmune gastritis, non-segmental vitiligo or celiac disease), patients were divided into group 1 (chronic autoimmune gastritis positive, *n* = 64), group 2 (non-segmental vitiligo positive, *n* = 24), and group 3 (celiac disease positive, *n* = 15). At least one of the four THAb was detected in 45 PAS-3 patients (42.1%) and 28 controls (31.8%, *P* = 0.14), with similar rates in the three PAS-3 groups. The rates of T3Ab, T4Ab, and T3 + T4Ab were similar in groups 1 and 2, while in group 3, T3Ab was undetected (*P* = 0.02). In PAS-3 patients, the rate of levothyroxine treatment was greater in THAb-positive patients compared to THAb-negative patients (76.7 vs. 56.1%, *P* = 0.03, RR = 1.4, 95% CI 1.03–1.81). Not unexpectedly, levothyroxine daily dose was significantly higher in group 1 and group 3, namely in patients with gastrointestinal disorders, compared to group 2 (1.9 ± 0.4 and 1.8 ± 0.3 vs. 1.5 ± 0.2 μg/kg body weight, *P* = 0.0005 and *P* = 0.004). Almost half of PAS-3 patients have THAb, whose repertoire is similar if chronic autoimmune gastritis or celiac disease is present. A prospective study would confirm whether THAb positivity predicts greater likelihood of requiring levothyroxine treatment.

## Introduction

Polyglandular autoimmune syndromes (PAS) are defined by the coexistence of different clusters of autoimmune diseases (1). Neufeld and Blizzard (2) originally defined PAS-3 as the association between one autoimmune thyroid disease (AITD) and one or more non-thyroidal autoimmune diseases (NTAID) other than Addison’s disease and hypoparathyroidism, since these two last diseases identify PAS-1 and PAS-2, respectively (1, 3). NTAID included in PAS-3 were type 1 diabetes mellitus (PAS type 3A); autoimmune chronic atrophic gastritis and pernicious anemia (type 3B); and vitiligo, alopecia, and myasthenia gravis (type 3C) (2). Subsequently, Betterle defined PAS-3 based on the different organ-specific and non-organ-specific NTAID that are associated with AITD (Hashimoto’s thyroiditis, idiopathic myxedema, symptomless autoimmune thyroiditis, Graves’ disease, and thyroid-associated ophthalmopathy) (4, 5). PAS-3A includes autoimmune endocrine diseases (type 1 diabetes mellitus, lymphocytic hypophysitis, premature ovarian failure, and Hirata’s disease); PAS-3B includes gastrointestinal autoimmune diseases (chronic atrophic gastritis, pernicious anemia, celiac disease, and chronic inflammatory bowel diseases) and hepatic autoimmune diseases (autoimmune hepatitis, primary biliary cirrhosis); PAS-3C comprises skin autoimmune diseases (vitiligo, alopecia, and urticaria), and nervous system autoimmune diseases (myasthenia gravis, multiple sclerosis, and stiff-man syndrome); PAS-3D includes collagen diseases (lupus erythematosus and its variants, rheumatoid arthritis, seronegative arthritis, systemic sclerosis, and mixed connective tissue disease), Sjogren syndrome, vasculitis, and hematologic autoimmune diseases (Werlhof’s syndrome and antiphospholipid syndrome) (4, 5).

The most common AITD is Hashimoto’s thyroiditis, also known as chronic lymphocytic thyroiditis. Like other autoimmune endocrine diseases, the prevalence of Hashimoto’s thyroiditis has shown a remarkable upward trend over the last decades (6, 7). The hallmarks of Hashimoto’s thyroiditis are the hypoechoic ultrasound pattern of thyroid and the high rate of positivity of thyroid autoantibodies (namely, antibodies directed against thyroglobulin and thyroperoxidase) (6). Interestingly, a decline in prevalence and serum levels of thyroid autoantibodies was noticed over the last decades in patients with Hashimoto’s thyroiditis, together with a specular increase in the prevalence of thyroid hormones autoantibodies (THAb) (6). Indeed, in those patients, the rate of THAb positivity increased by eightfold (from 5 to 40%) over the period 1975–2005 (6, 8). THAb are found in patients with Graves’ disease in around one-third of cases (8, 9). Also, the real prevalence of THAb may be underestimated because they are not routinely assayed and can appear transiently in serum (8, 10). THAb, which can be found in approximately 1% of the general population, are directed against T3, T4, or both, and may belong to IgM, IgG, or both classes (8, 11). Our group has shown that at least one THAb can be found in sera of patients with NTIAD even more frequently compared to AITD, regardless of thyroglobulin and/or thyroperoxidase antibodies status. For instance, in primary Sjogren syndrome or rheumatoid arthritis, the rate of THAb positivity was 55 or 40%, respectively, and has increased steadily over the last years (8, 9). Recently, the highest rates of THAb positivity were found in patients with type 1 diabetes mellitus or vitiligo, since approximately all of them carried at least one THAb (12–14).

Because never studied before, we evaluated the prevalence and repertoire of THAb in PAS-3 patients.

## Subjects and Methods

This study complies with the Declaration of Helsinki. Informed consent has been obtained from each patient after full explanation of the purpose and nature of all procedures used. Since this was not an interventional study, we did not need any approval by the Internal Review Board.

Our cohort consists of 107 consecutive PAS-3 patients (90 females, 17 males, F:M ratio = 5.3:1), aged 15–82 years at first observation and associated blood sampling. All patients had one AITD [Hashimoto’s thyroiditis (*n* = 103), Graves’ disease (*n* = 4)] associated with at least one NTAID. On the basis of the three most prevalent NTAID (chronic atrophic gastritis, non-segmental vitiligo, and celiac disease), we divided the 107 patients into three mutually exclusive groups. In group 1 patients (*n* = 66; 61.7%), HT coexisted with chronic atrophic (autoimmune) gastritis alone (*n* = 42) or chronic atrophic gastritis plus at least another NTAID (*n* = 24) (Table 1). In group 2 patients (*n* = 26; 24.3%), HT coexisted with non-segmental vitiligo alone (*n* = 24) or non-segmental vitiligo plus at least another NTAID (*n* = 2) (Table 1). Finally, in group 3 patients (*n* = 15; 14.0%), HT coexisted with only celiac disease and no patient presented with celiac disease plus another NTAID (Table 1).

**Table 1:** Demographics of patients and controls according to AITD and NTAID.

Group AITD Selective NAITD	1 (*n* = 66) HT or GD^a^ CAG	2 (*n* = 26) HT NSV	3 (*n* = 15) HT or GD^a^ CD	All (*n* = 107) HT + GD CAG + NSV + CD
**Other NAITD**				
Only one (selective)	42	24	15	76
Two	19	2	0	23
Three	5	0	0	8
Four or more	0	0	0	0
**Most frequent NAITD**				
Pernicious anemia	13	0	0	13
Rheumatoid arthtritis	3	0	0	0
Sjogren syndrome	3	0	0	0
**Other indices**				
Age, years	55.4 ± 13.6	45.5 ± 14.1	37.4 ± 12.2	50.5 ± 15.0
Gender (females, males) (F:M ratio)	61, 5 (12.2:1)	16, 10 (1.6:1)	13, 2 (6.5:1)	90, 17 (5.3:1)
Under L-T4 replacement	*n* = 45 (68.2%)	*n* = 16 (61.5%)	*n* = 6 (40%)	*n* = 67 (62.6%)

*Not to overload the table we have shown only the most frequent NTAID, namely, those in ≥3 patients*.

*AITD, autoimmune thyroid disease; CAG, chronic autoimmune gastritis; CD, celiac disease; GD, Graves’ disease; HT, Hashimoto’s thyroiditis; NAITD, non-autoimmune thyroid disease; NSV, non-segmental vitiligo*.

*^a^Only four patients had GD as AITD, two of them were in group 1 and another two in group 3*.

The control group was previously studied to also serve as a control group in another setting (9). It consisted of a previously cohort of 88 Hashimoto’s thyroiditis patients (77 women, 11 men, F:M ratio = 7:1; age 42 ± 9 years) who had never been treated with levothyroxine (L-T4) and, importantly, had no NTAID.

### Diagnosis of Autoimmune Diseases

Hashimoto’s thyroiditis was defined by hypoecheoic and inhomogeneous echopattern with or without positivity of either or both thyroid autoantibodies (thyroglobulin antibodies and thyroperoxidase antibodies) (6, 15). Diagnosis of chronic atrophic gastritis was based on parietal cell antibodies positivity and histological confirmation (i.e., focal or complete loss of oxyntic glands and/or replacement by metaplastic pyloric or intestinal glands) (16). According to the Vitiligo European Task Force definition (17), non-segmental vitiligo was defined by the presence of white patches of skin, generally symmetrical and growing in size. Celiac disease was supported by positivity of serum transglutaminase antibodies (IgG and IgA) and/or endomysial antibodies (IgG and IgA). Celiac disease was histologically confirmed after duodenum and jejunum biopsies showing various degrees of intestinal atrophy and crypt hyperplasia according to the Marsh classification, as modified by Oberhuber (18).

### Thyroid Hormone Autoantibodies (THAb) Assay

We assayed serum THAb (T3IgM, T3IgG, T4IgM, and T4IgG) by a previously described radioimmunoprecipitation technique (10, 12, 19). Five hundred microliters of serum, which had been stored at –20°C, were incubated with 0.5 μCi [^125^I]T3 or [^125^I]T4 (Johnson and Johnson, Milan, Italy) for 60 min at 23°C. Twenty microliters of this mixture were incubated with 150 µL solution containing antihuman IgM or antihuman IgG serum (Sigma-Aldrich, Milan, Italy) at a concentration of 0.5% for 24 h at 4°C. Both antihuman IgM and antihuman IgG serum had been prediluted 1:10 with saline containing BSA. The mixture obtained was then centrifuged at 2,000 × *g* for 20 min, and the supernatant was aspirated. T3Ab and T4Ab were assayed in two different runs. We also assayed THAb in negative and positive controls, namely sera negative for all four types of THAb or sera positive for one type of THAb (T3IgM, T3IgG, T4IgM, or T4 IgG). Each serum was analyzed in duplicate for each THAb. THAb positivity was defined by a proportion of the [^125^I]T3 or [^125^I]T4 immunoprecipitated by the corresponding antiserum >3.9%, >3.6%, >3.4%, or >3.9% for T3IgM, T3IgG, T4IgM, or T4IgG, respectively (10, 12, 19). In case of borderline values, assay was repeated.

### Statistics

Data are reported as mean ± SD. Comparisons between proportions were handled with the χ^2^-test or the Fisher’s exact test as appropriate. Comparisons between continuous variables were handled with the Mann–Whitney test or the Kruskal–Wallis test as appropriate. Level of significance was set at 0.05.

## Results

### Demographics

The mean age at first observation in the 107 PAS-3 patients was 50.5 years (range 15–82 years). Group 1 patients were significantly older compared with group 2 (55.4 ± 13.6 vs. 45.5 ± 14.1 years, *P* = 0.004) and group 3 (55.3 ± 13.8 vs. 37.4 ± 12.2 years, *P* < 0.0001) patients, while group 3 patients were younger, although not significantly, compared to group 2 patients (*P* = 0.06) (Table 1). Not unexpectedly patients with chronic autoimmune gastritis were the oldest, as this autoimmune disease peaks some decades later (16) in contrast with non-segmental vitiligo (17) and celiac disease (20).

The female-to-male ratio (F:M) was significantly greater in group 1 than in group 2 (61:5 or 12.2:1 vs. 16:10 or 1.6:1, *P* = 0.0008).

Excluding the four patients with Graves’ disease, at first observation, 67/107 HT patients (62.6%) were under replacement L-T4 therapy, with similar intragroup rates (group 1, 45/66, 68.2%; group 2, 16/26, 61.5%; group 3, 6/15, 40%).

### Prevalence and Repertoire of THAb

All data are summarized in Table 2. Overall, THAb were detected in 45/107 patients (42.1%), without significant differences between groups (43.9, 42.3, and 33.3% in groups 1, 2 and 3, respectively).

**Table 2:** Prevalence and repertoire of THAb.

THAb				AITD + CAG *n* = 66 Group 1	AITD + NSV *n* = 26 Group 2	AITD + CD *n* = 15 Group 3	PAS-3 *n* = 107 All	HT^a^ *n* = 88 Controls
**T3Ab**		**T4Ab**						
**IgM**	**IgG**	**IgM**	**IgG**					
No	No	No	No	37 (56.1%)	15 (57.7%)	10 (66.7%)	62 (57.9%)	60 (68.2%)
At least one of the four +ve				29 (43.9%)	11 (42.3%)	5 (33.3%)	45 (42.1%)	28 (31.8%)
**Single THAb**				25 (37.9%)	9 (34.6%)	5 (33.3%)	39 (36.4%)	19 (21.6%)
+	No	No	No	13 (19.7%)	3 (11.6%)	0	16 (14.9%)	2 (2.3%)
No	+	No	No	5 (7.6%)	3 (11.6%)	0	8 (7.5%)	6 (6.8%)
No	No	+	No	2 (3.0%)	0	4 (26.7%)	6 (5.6%)	8 (9.1%)
No	No	No	+	5 (7.6%)	3 (11.5%)	1 (6.7%)	9 (8.4%)	3 (3.4%)
**Double THAb**				3 (4.5%)	2 (7.7%)	0	5 (4.7%)	9 (10.2%)
+	+	No	No	2 (3.0%)	1 (3.8%)	0	3 (2.8%)	5 (5.7%)
+	No	+	No	0	0	0	0	1 (1.1%)
No	No	+	+	0	0	0	0	1 (1.1%)
No	+	+	No	0	0	0	0	1 (1.1%)
No	+	No	+	0	0	0	0	0
+	No	No	+	1 (1.5%)	1 (3.8%)	0	2 (1.9%)	1 (1.1%)
**Triple THAb**				1 (1.5%)	0	0	1 (0.9%)	0
+	+	+	No	1 (1.5%)	0	0	1 (0.9%)	0
+	No	+	+	0	0	0	0	0
No	+	+	+	0	0	0	0	0
+	+	No	+	0	0	0	0	0
**Quadruple THAb**								
+	+	+	+	0	0	0	0	0
**Based on hormone bound**								
T3 only (IgM, IgG, or both)				20 (30.3%)	7 (26.9%)	0	27 (25.2%)	13 (14.8%)
T4 only (IgM, IgG, or both)				7 (10.6%)	3 (11.5%)	5 (33.3%)	15 (14.0%)	12 (13.6%)
T3 and T4 (IgM, IgG, or both)				2 (3.0%)	1 (3.8%)	0	3 (2.8%)	3 (3.4%)
All absent				37 (56.1%)	15 (57.7%)	10 (66.7%)	62 (57.9%)	60 (68.2%)
**Based on Ig class**								
IgM only (T3, T4, or T3 + T4)				15 (22.7%)	3 (11.5%)	4 (26.7%)	22 (20.6%)	11 (12.5%)
IgG only (T3, T4, or T3 + T4)				10 (15.1%)	6 (23.1%)	1 (6.7%)	17 (15.9%)	9 (10.2%)
IgM and IgG (T3, T4, or T3 + T4)				4 (6.1%)	2 (7.7%)	0	6 (5.6%)	8 (9.1%)
All absent				37 (56.1%)	15 (57.7%)	10 (66.7%)	62 (57.9%)	60 (68.2%)

*For abbreviations, see Table 1 footnote*.

*^a^At time of blood sampling, there was no non-thyroid autoimmune disease associated with HT*.

Single THAb were the most prevalent (36.4%), again with no intergroup differences (33.3–37.9%), whereas quadruple THAb were always absent. Within the single THAb category, the four types of THAb were distributed similarly in groups 1 and 2 (df = 3, χ^2^ = 2.2, *P* = 0.53), but dissimilarly between groups 1 and 3 (df = 3, χ^2^ = 14.4, *P* = 0.002) or between groups 2 and 3 (df = 3, χ^2^ = 10.7, *P* = 0.01). Because of very low or absent types of THAb in the double, triple, or quadruple categories, no intergroup statistics was possible (Table 2).

Concerning categorization based on the thyroid hormones bound (i.e., T3 only, T4 only, and T3 plus T4), groups 1 and 2 did not differ. In contrast, because of the selective binding of T4 by THAb in group 3 patients, this group differed from both group 1 (df = 2, χ^2^ = 10.8, *P* = 0.005) and group 2 (df = 2, χ^2^ = 7.3, *P* = 0.03). In regard to categorization based on the immunoglobulin classes of THAb (i.e., IgM only, IgG only, and IgM plus IgG), group 3 did not differ from the other two groups (χ^2^ = 1.6, *P* = 0.45 vs. group 1; χ^2^ = 4.0, *P* = 0.13 vs. group 2), with groups 1 and 2 not differing either (χ^2^ = 2.0, *P* = 0.37) (Table 2).

Six of the 13 group 1 patients with pernicious anemia carried one THAb (three patients carried T4IgG, two patients T3IgM, and one patient T4IgM). Only one patient of the three with rheumatoid arthritis or Sjogren syndrome was THAb positive (T3IgM).

### Prevalence and Repertoire of THAb in PAS-3 Patients Compared to Controls

Table 2 also compares the repertoire of THAb in PAS-3 patients with that of the HT only patients. Even though the rate of THAb positivity (i.e., positivity for at least one THAb) was similar (42.1 vs. 31.8%, *P* = 0.14), the four types of THAb were dissimilarly distributed in PAS-3 patients and controls (df = 3, χ^2^ = 8.6, *P* = 0.03), the greatest difference being between group 1 patients and controls (df = 3, χ^2^ = 11.7, *P* = 0.009). Concerning categorization based on the thyroid hormones bound, in PAS-3 patients, the most prevalent THAb were against T3 (25.2%) compared to those against T4 (14%) or both T3 and T4 (2.8%). In contrast, the corresponding rates in controls were 14.8, 13.6, and 3.4% (*P* = 0.51) (Table 2). PAS-3 patients and controls did not differ either in the repertoire of THAb based on immunoglobulin classes (*P* = 0.27) (Table 2).

### Association of THAb with Number of NTAID

The average number of NTAID was the highest in group 1 patients, the lowest in group 3 patients, and intermediate in group 2 patients (1.4 vs. 1.0 vs. 1.1, *P* = 0.0009). Indeed, around one-third (36.4%) of group 1 patients had at least two NTAID, in contrast with 2/26 (7.7%) patients in group 2 and none in group 3 (36.4 vs. 7.7% vs. 0, df = 2, χ^2^ = 13.9, *P* = 0.0009).

### Association of THAb with Replacement L-T4 Therapy in PAS-3 Patients

Seventy patients (65.4%) were on L-T4 replacement therapy at the time of blood sampling. THAb-positive PAS-3 patients were more likely to be on L-T4 compared to THAb-negative patients (76.7 vs. 56.1%, *P* = 0.03, RR = 1.4, 95% CI 1.03–1.81). Furthermore, there was no difference in the frequency of replacement therapy considering either thyroid hormones bound or classes of immunoglobulin (data not shown). In the remaining 37 patients who did not take L-T4, serum TSH levels were similar in THAb-positive and THAb-negative patients (3.4 ± 1.8 vs. 3.4 ± 3.0 mU/L, *P* = 0.51).

The mean L-T4 daily dose was 1.8 ± 0.4 μg/kg body weight and was greater in group 1 and group 3 patients (1.9 ± 0.4 and 1.8 ± 0.3 μg/kg body weight) compared to group 2 patients (1.5 ± 0.2 μg/kg body weight, *P* = 0.0005 and *P* = 0.004, respectively). L-T4 daily dose did not change significantly according to THAb status (THAb positive 1.7 ± 0.3 μg/kg body weight vs. THAb negative 1.9 ± 0.5 μg/kg body weight, *P* = 0.12).

## Discussion

In this study, we have shown that in PAS-3 patients, THAb are found frequently, namely in 4 out of 10. By comparing data of PAS-3 patients with only Hashimoto’s thyroiditis (whose THAb positivity rate is three out of 10), we infer, as mentioned earlier, that the presence of NTAID in the background of Hashimoto’s thyroiditis adds little influence on the propensity to form THAb, with negligible influence of associated NTAID on thyroid hormones bound by THAb and classes of immunogloblulin.

Detection of one or more THAb in PAS-3 patients cannot be explained only by the presence of a mere genetic background, namely proclivity of these patients to produce antibodies against autoantigens. THAb are autoantibodies directed against epitopes of thyroglobulin containing iodinated tyrosines, which can form T3 only, T4 only, or both T3 and T4 (11). As demonstrated previously (10) in Hashimotos’ thyroiditis patients who were THAb negative prior to undergo diagnostic fine-needle aspiration biopsy (FNAC), the appearance in serum of THAb is preceded by the FNAC-elicited release of iodinated and heterologous moieties of thyroglobulin that were absent prior to FNAC. Leakage of such thyroglobulin molecules may occur naturally in the time course of Hashimotos’ thyroiditis, for instance during phases of aggravation of the autoimmune inflammation (8). Inflammatory cytokines may disclose hidden thyroglobulin epitopes, which are capable to elicit autoantibodies production by B-lymphocytes that infiltrate the thyroid (8). Post-translational modifications of thyroglobulin may increase its antigenicity by better exposing the said hormonogenic epitopes (21). For instance, in patients with type 1 diabetes mellitus, glycosylated thyroglobulin might elicit THAb synthesis with high frequency (12). One possible explanation for the detection of THAb in NTAID *per se* is that a very initial autoimmune inflammation of the thyroid is already present in NTAID. Indeed, the early stages of lymphocytic thyroiditis are characterized by serum reactivity against either thyroid hormone, even though thyroglobulin antibodies that can be assayed by conventional methods are undetectable (22). Another explanation is that material leaking from the inflamed peripheral tissues of NTAID patients (e.g., joints, stomach, intestinum, and skin) and, regardless of sharing molecular mimicry with thyroglobulin, binds thyroid hormones, thus forming novel iodinated autoantigens (8).

Concerning the THAb repertoire, double or triple THAb positivity was much less common compared to single positivity. Particularly, of the 52 THAb detected in the 45 THAb-positive PAS-3 patients, three-fourth (39/52, 75%) were directed against either T3 or T4. Concerning hormone specificity of THAb, while in groups 1 and 2, T3Ab outnumbered T4Ab, and in group 3, the distribution was inverted. This difference resembled that found in patients with type 1 diabetes mellitus, in which the rate of T3Ab or T4Ab positivity was 38.5% (20/52) or 27.1% (12/52) (12). Interestingly, no patient of this cohort had type 1 diabetes mellitus. In patients with only Hashimoto’s thyroiditis, T3Ab and T4Ab were equally distributed (Table 2). Classes of THAb varied remarkably among groups, with a preponderance of T3IgM or T4IgM in group 1 or group 3, and similar rates of T3IgM, T3IgG, and T4IgG in group 2. Thus, overall, primary immune response (i.e., IgM) prevailed over a stable secondary immune response (i.e., IgG).

Concerning additional NTAID, they were more common in group 1. This was not unexpected, since those patients were older than group 2 and group 3 patients (see above). The most common additional NTAID was pernicious anemia, which was found in 1/4 patients with chronic atrophic gastritis. Pernicious anemia results from vitamin B_12_ deficiency and may occur in patients with advanced chronic inflammation of the gastric corpus. In up to 80% of patients with pernicious anemia, autoantibodies against intrinsic factor, which impair vitamin B_12_ absorption, are found (16, 23).

THAb positivity was associated with a greater risk of hypothyroidism (RR = 1.4, 95% CI 1.03–1.81) regardless of the hormone bound or the class of immunoglobulin. Indeed, more than 3/4 THAb-positive PAS-3 patients had required L-T4 as thyroid replacement, in contrast with around half of THAb-negative PAS-3 patients. Not unexpectedly patients with the greatest requirements of L-T4 were those with gastric autoimmune gastritis or celiac disease, two disorders that are associated with impaired intestinal absorption of L-T4 (24–27). For instance, patients with chronic atrophic gastritis need a 27% increase in L-T4 dose in order to achieve target serum TSH levels (24, 28). Furthermore, atrophic gastritis can be associated with a long-standing *Helicobacter pylori* infection in up to 30% of cases (16, 29). Compared to *H. pylori*-negative patients, those with a concurrent *H. pylori* infection need a greater increase in L-T4 dose (+34 vs. +24%) (24).

One limitation of this otherwise novel study is its cross-sectional design. Therefore, we could not evaluate (i) the occurrence of other NTAID over time based on THAb status, (ii) the switch of immune response from IgM to IgG, and (iii) changes of the rate of hypothyroidism over time based on THAb status.

In conclusion, almost half of PAS-3 patients have THAb, whose repertoire is similar in the group with autoimmune gastritis and the group with celiac disease. A prospective study would confirm whether THAb positivity predicts a greater likelihood of requiring L-T4 treatment over the years.

## Ethics Statement

This study complies with the Declaration of Helsinki. Informed consent has been obtained from each patient after full explanation of the purpose and nature of all procedures used.

## Author Contributions

SB conceived the study. MG and MM performed the THAb assays. RV, MGS, CV, MS, FB, MC, and SB analyzed the data. RV, MGS, CV, MC, and SB drafted the manuscript. All the authors contributed to the final revision of the manuscript.

## Conflict of Interest Statement

The authors declare that the research was conducted in the absence of any commercial or financial relationships that could be construed as a potential conflict of interest.

## Abbreviations

AITD, autoimmune thyroid disease; L-T4, levothyroxine; NTAID, non-thyroidal autoimmune disease; PAS, polyglandular autoimmune syndrome; THAb, thyroid hormones antibodies.
